# Cost-Effectiveness of Alternative Strategies for Annual Influenza Vaccination among Children Aged 6 Months to 14 Years in Four Provinces in China

**DOI:** 10.1371/journal.pone.0087590

**Published:** 2014-01-31

**Authors:** Lei Zhou, Sujian Situ, Zijian Feng, Charisma Y. Atkins, Isaac Chun-Hai Fung, Zhen Xu, Ting Huang, Shixiong Hu, Xianjun Wang, Martin I. Meltzer

**Affiliations:** 1 Public Health Emergency Center, Chinese Center for Disease Control and Prevention, Beijing, China; 2 U.S. Centers for Disease Control and Prevention, Beijing, China; 3 U.S. Centers for Disease Control and Prevention, National Center for Emerging and Zoonotic Infectious Diseases, Division of Preparedness and Emerging Infections, Atlanta, Georgia, United States of America; 4 Department of Epidemiology, Jiann-Ping Hsu College of Public Health, Georgia Southern University, Statesboro, Georgia, United States of America; 5 Key Laboratory of Surveillance and Early-warning on Infectious Disease, Division of Infectious Disease, Chinese Center for Disease Control and Prevention, Beijing, China; 6 Sichuan Center for Disease Control and Prevention, Chengdu, Sichuan, China; 7 Hunan Center for Disease Control and Prevention, Changsha, Hunan, China; 8 Shandong Center for Disease Control and Prevention, Jinan, Shandong, China; Fudan University, China

## Abstract

**Background:**

To support policy making, we developed an initial model to assess the cost-effectiveness of potential strategies to increase influenza vaccination rates among children in China.

**Methods:**

We studied on children aged 6 months to 14 years in four provinces (Shandong, Henan, Hunan, and Sichuan), with a health care system perspective. We used data from 2005/6 to 2010/11, excluding 2009/10. Costs are reported in 2010 U.S. dollars.

**Results:**

In comparison with no vaccination, the mean (range) of Medically Attended Cases averted by the current self-payment policy for the two age groups (6 to 59 months and 60 months to 14 years) was 1,465 (23∼11,132) and 792 (36∼4,247), and the cost effectiveness ratios were $ 0 (-11-51) and $ 37 (6-125) per case adverted, respectively. In comparison with the current policy, the incremental cost effectiveness ratio (ICER) of alternative strategies, OPTION One-reminder and OPTION Two-comprehensive package, decreased as vaccination rate increased. The ICER for children aged 6 to 59 months was lower than that for children aged 60 months to 14 years.

**Conclusions:**

The model is a useful tool in identifying elements for evaluating vaccination strategies. However, more data are needed to produce more accurate cost-effectiveness estimates of potential vaccination policies.

## Introduction

Influenza can increase acute respiratory infections and hospital admissions, imposing a significant burden of illness among vulnerable groups including children. The World Health Organization (WHO) and many countries recommend annual influenza vaccines for children [1]–[3]. In 2003, the Chinese Ministry of Health issued the first guidance on seasonal influenza vaccination [4]. Prior to the start of each influenza season since 2007, the Chinese Center for Disease Control and Prevention (China CDC) released annual seasonal influenza vaccination guidance recommending that children should be included in the priority population [5]–[8]. Nonetheless, the influenza vaccination rate among children in China remains low, in part due to a low level of available vaccine supply sufficient for 1.9% of the Chinese population [9].

China's influenza vaccination program is administered by the CDC system. Every year, local governments authorize local CDCs to purchase vaccine before the influenza season and deploy to vaccination clinics. These clinics are usually located in close proximity to the local CDC or other community health care centers, but not in hospitals. Patients have to pay for the vaccination, except in a few cities such as Beijing. The influenza vaccination policy has not been modified for many years because decision makers lack scientific evidence on the impact of alternative strategies.

The objective of this study is to present for public health policy makers, an initial model, using both available data and assumptions, to evaluate the cost-effectiveness of the current influenza vaccination policy in children as well as other alternative influenza vaccination strategies in China.

## Methods

We built a spreadsheet-based model to evaluate the cost-effectiveness of current seasonal influenza vaccination policies in children aged 6 months to 14 years in four provinces (Shandong, Henan, Hunan, and Sichuan) in China. We also developed a methodology to evaluate the cost-effectiveness of using alternative strategies to increase the influenza vaccination coverage rate among children in China. All analyses are from the health care system perspective with the government as the payer. Our study focused on patients who attended government-run hospitals and clinics. Costs are reported in 2010 U.S. dollars. We did not discount any cost or health outcomes as each influenza season lasts less than one year.

### Variables to estimate the number of cases

The population (P) for the model was census data for calendar year 2009 [10]. Two age groups of children were analyzed, 6 to 59 months and 60 months to 14 years. See Table S1 in Appendix S1 for specific provincial population numbers.

We used five years of epidemiological data from the season 2005/06 to 2010/11 according to Chinese Influenza-Like-Illness (ILI) sentinel surveillance system, excluding the 2009/10 season which was influenza pandemic, causing an extraordinary public health response with large vaccination coverage [11]. See Table S2 in Appendix S1 for specific seasonal and provincial numbers.

**Number of outpatients and inpatients:** We estimated the number of confirmed influenza outpatients among children using the following equation:

# confirmed influenza outpatients  =  rate of outpatient visits (p1)×rate of ILI cases in outpatients (p2) ×influenza positive rate among ILI (p3) ×population (P)

Data for the rate of outpatient visits for all causes (p1) (Table 1) came from the 4^th^ National Health Services Survey [12], which asked for respondents' outpatient visits in the previous two weeks. Assuming the influenza season lasted for 24 weeks, we then multiplied the rate of outpatient visits in 2 weeks by 12 to get the overall rate of the influenza season.

**Table 1:** Epidemiology input data of model.

Parameter and definition		Age group	Mean or Median (range)	Source
P	Population	6–59 ms	14,301,091	Statistic yearbook in China, 2009 [10]
		60 ms-14 yrs	45,737,342	
p1	Number of outpatients of age specific visit per 100 persons of all age for any cause in influenza season (6 months)	6–59 ms	297.60	4^th^ national health service research in 2008 [12]
		60 ms-14 yrs	109.20	
p2	ILI of age specific per 100 outpatients of all age	6–59 ms	1.58 (0.22, 3.30)	ILI sentinel surveillance, CCDC (from 05/06 to 10/11 except 09/10 season)
		60 ms-14 yrs	0.55 (0.16, 0.99)	
p3	Influenza positive per 100 ILI with lab test of age specific	6–59 ms	9.08 (6.95, 11.30)	ILI sentinel surveillance, CCDC (from 05/06 to 10/11 except 09/10 season)
		60 ms-14 yrs	17.47 (14.77, 22.72)	
p4	ILI outpatients of age specific per SARI hospitalization in same SARI sentinel hospital	6–59 ms	5.63 (0.32–593.63)	Data of cost survey in three SARI sentinel hospitals in 2011
		60 ms-14 yrs	8.58 (0.75–941.00)	
p5	Number of influenza positive per 100 SARI cases	6 ms-14 yrs	14.97	Data of cost survey in three SARI sentinel hospitals in 2011
VCR	Influenza vaccination coverage rate (%)	6–59 ms	11.85 (1.18, 37.69)	(1)VCR of 05/06-08/09 are assumed as ratio of sales vs pop, and VCR of two age groups are assumed same; (2)VCR of 10/11 and 11/12 were from telephone survey in five provinces conducted by CCDC in 2011
		60 ms-14 yrs	10.81 (1.18, 39.09)	
VE	Influenza vaccine effectiveness (%)	6 ms-14 yrs	61 (52, 68)	A.S. Monto et al, 2009 [15], D.M. Skowronski et al, 2007 [16], [17] Eelko Hak, et al, 2000 [18], Steens A, et al, 2011 [19]

Footnotes: ms: months; yrs: years. See Table S2 in Appendix S1 for specific seasonal and provincial numbers.

The rate of ILI (p2) and influenza positive rate among ILI (p3) were from the Chinese ILI sentinel surveillance system, which records demographic, clinic visit and laboratory testing data by specific province.

We used the following equation to calculate the number of confirmed influenza inpatients:

# confirmed influenza inpatients =  ((rate of outpatient visits (p1) × rate of ILI cases in outpatients (p2))/rate of ILI outpatients per Severe Acute Respiratory Infection (SARI) hospitalization (p4)) ×influenza positive rate among SARI cases (p5) ×population (P)

We used Chinese sentinel SARI surveillance data, from Oct 1, 2010 to Mar 31, 2011, to calculate the rate of ILI outpatients per SARI inpatient (p4) (Table 1). The SARI surveillance hospitals were also the ILI surveillance hospitals. Patients who met the SARI definitions [13] were eligible for enrollment and nasopharyngeal and throat swabs were collected for influenza viruses testing as described previously [13], [14]. Once each SARI case is tested for influenza virus, we then have the influenza positive rate per 100 SARI cases (p5).

**Number of cases adverted:** We collected the age and province specific influenza vaccination coverage rate for seasons 2010/11 and 2011/12 from telephone surveys conducted by China CDC in 2011 (Table 1 and Table S3 in Appendix S1) [15]. We also used the annual sales of influenza vaccine to estimate the coverage rate for season 2005/06 to 2008/09, and assumed the coverage rate was the same for children in both age groups.

The influenza vaccine effectiveness by age group was collected from published literature [16]–[20]. The mean was 61% with range of 52% to 68% (Table 1).

In this study we used the health outcome of Medically Attended Cases (MAC), excluding those who do not seek medical care in public health facilities.

We applied the following equation to generate the number of cases averted:

# cases averted (both outpatient and inpatient)  =  (# confirmed influenza outpatients + # confirmed influenza inpatients)/(1 – % vaccine coverage×% vaccine effectiveness) ×% vaccine coverage×% vaccine effectiveness

### Variables used to estimate the economic impact of vaccination

**Program and vaccine cost:** Data were not available on the programmatic cost of the influenza vaccination program in China. However, the Expanded Program of Immunization (EPI) system shares the same management system with the influenza vaccination program and those data are documented in previous reports [21]. The programmatic cost of the EPI system per dose per child was $ 1.69, including staff payment at provincial, prefecture and county level, program operation (i.e. advertisement, training, and monitoring), logistics, and cold-chain (Table 2).

**Table 2:** Cost input data of model.

Parameter and definition	Group	Value (US$)	Source
Mean cost of EPI system per child per dose	Total programmatic cost	1.69	WZ Yu, et al, 2006 [19]
	staff payment	0.52	
	program operation	0.73	
	logistics	0.35	
	cold-chain	0.09	
Cost of influenza vaccine per dose	Government purchase price	3.95	Sichuan's data, authors' unpublished data
Vaccination fee	Vaccination fee to parents	5.61	Sichuan's data, authors' unpublished data
Net cost of influenza vaccination program to government per child per dose	Current policy	0.03	Model calculation
Cost of confirmed influenza outpatient aged 6ms-14 yrs	Direct medical cost	11.28	Zhou L, et al, 2013 [13]
Cost of confirmed influenza inpatient aged 6ms-14 yrs	Direct medical cost	284.44	

Footnotes:

The original currency was CNY and has been converted to USD using exchange rate in 2010 (100 US = 677 CNY).

We only included the cost that was reimbursed by the health insurance, and did not include the proportion that was paid by parents.

The estimated reimbursement rate from the survey was 19.25% for children. Therefore, the health care system paid $2.17 per outpatient case and $54.67 per inpatient case.

In China, influenza vaccines are purchased from both domestic and international suppliers. We assumed that vaccine cost was the same as the purchase price. In season 2010/11, the mean (range) government purchase price of influenza vaccine for children was $ 3.20 (2.89–3.38) for domestic suppliers and $ 6.59 (6.43–6.75) for international suppliers.

Based on sales from 2010/11, we estimated that 78% of the vaccines purchased were from domestic suppliers and 22% were from international suppliers [9]. Therefore our weighted average of government purchase price was $ 3.95.

Under the current policy, patients pay $ 4.52 (3.94–4.87) for domestic vaccine and $ 9.49 (8.57–9.65) for vaccines produced by international companies (authors' unpublished data), with a weighted average of $ 5.61 in the four provinces.

We used the following equation to determine the cost of the influenza vaccination program to the government (per child per dose) under the current self-payment policy:

Cost of influenza vaccination program to the government (per child per dose)  =  Programmatic cost per child per dose + cost of influenza vaccine per dose to government - price paid by parents per dose  =  $1.69 + $3.95 − $5.61  =  $ 0.03.

**Cost of illness (Outpatient and inpatient costs):** We used the health care system perspective with the government as the payer. Our study focused on patients who attended government-run hospitals and clinics, and we only included the cost borne by the health care system. We did not include patient co-payments, direct non-medical cost and lost productivity.

The cost of confirmed influenza outpatient and inpatient cases for children aged 6 months to 14 years was estimated $ 11.28 and $ 284.44, respectively. The estimated reimbursement rate from the survey was 19.25% for children. Parents paid the remainder. Therefore, the health care system paid $2.17 per outpatient case and $54.67 per inpatient case. The estimated cost and reimbursement rate was based on a survey [13].

### Cost-effectiveness analysis of the current policy

Expected costs, outcomes, and cost effectiveness ratios (CERs) were calculated in the spreadsheet model for each of the four provinces and the two age groups from season 2005/06 to 2010/11, excluding season 2009/10. CERs were reported as cost per case averted for the current influenza vaccination program compared with no vaccination.

We used historical data on clinical visits and vaccination coverage rates in the four provinces as the best available data to model the natural fluctuation of influenza prevalence and vaccination coverage rate.

The equation below was used to calculate the CERs:

$ Cost per case adverted  =  Net cost/# cases averted  =  ($ cost of influenza vaccination program –$ health care cost saved)/# cases averted

### Evaluation of alternative strategies to increase vaccination rate

We also used this model to evaluate the cost-effectiveness of alternative strategies.

**Figure 1:**
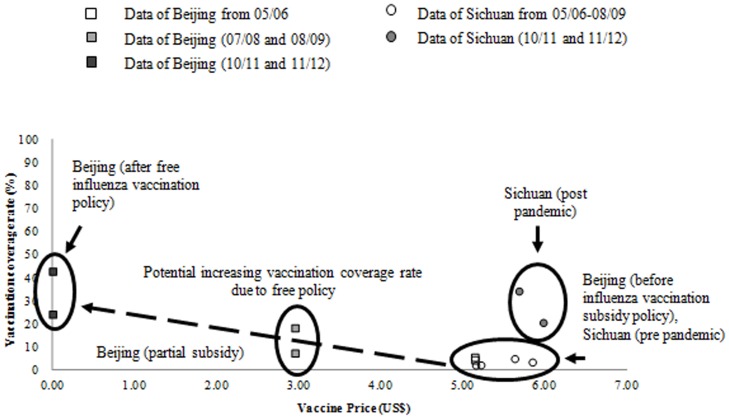
Impact on demand due to price of vaccine change to the parents. This figure used data of vaccination coverage rate and vaccine price to the parents in Beijing and Sichuan Province from season 05/06 to 11/12, excluding 09/10 the pandemic season. Beijing initiated an influenza vaccination subsidy policy for children and the elderly in 2007 and 2008, and has provided free influenza vaccine for children and the elderly since 2009.

**Price and demand curve:** To explore the impact on demand due to change in vaccine price to parents, we drew a price and demand curve (Figure 1). Given that the Beijing public health authority began to subsidize influenza vaccination for school children in 2007 and to provide free influenza vaccination to school children in 2009 [22], we compared vaccination coverage rates in Beijing before and after subsidy and free vaccination. We also analyzed vaccine coverage rates in Sichuan pre- and post-pandemic to illustrate the effects of other factors, since there was no change in price.

**OPTION description and assumptions:** Experience from other countries suggests that measures such as providing vaccination reminders, sending free vaccination vouchers to parents, and expanding vaccination sites could increase influenza vaccination rates [23]–[25]. We constructed two alternative OPTIONs for increasing vaccination rates based on these experiences with adjustment for the Chinese situation.

OPTION One is a telephone reminder to parents of targeted children for influenza vaccination. For example, staff in the EPI system can call parents before influenza season, reminding them to bring their children for influenza vaccination. To capture different levels of impact by this measure, we assumed this could increase the vaccination rate by 5%, 10%, and 15%. We also assumed that the current system could absorb a 5% and 10% increase in vaccination rate, and the program cost per child was assumed to be the same as prior. When the vaccination rate increased by 15%, we assumed that the current system would require additional resources to meet the demand, so the programmatic cost per child would be 1.5 times prior cost (Table 3).

**Table 3:** Impact of illustration potential options to increase influenza vaccination rate among children (6 months to 14 years) in China.

Measures	Input variable	Value	Source
OPTION One: recall/text message reminder	Impact of increasing vaccination rate (%)	5, 10, 15	Eelko Hak, et al, 2000 [17] and assumption
	Cost of EPI system (US$)	Prior: $1.69	Assumption
	If vaccination rate increases 5%	same with prior $	
	If vaccination rate increases 10%	same with prior $	
	If vaccination rate increases 15%	1.5 times of prior $	Assumption
	Cost of Recall (US$)	0.17	
	staff cost	0.14	
	Telephone charge	0.03	
OPTION Two: free vouchers and expanding vaccination sites	Impact of increasing vaccination rate (%)	10, 15, 25	Rancé F, et al, 2008 [22], Daley MF, et al, 2004 [23], Britto MT, et al, 2007 [24] and assumption
	Cost of EPI system (US$)	Prior: $1.69	Assumption
	If vaccination rate increases 10%	same with prior $	
	If vaccination rate increases 15%	1.5 times of prior $	
	If vaccination rate increases 25%	2.5 times of prior $	
	Cost of measure (US$)	0.73, 0.84, 1.18	
	Voucher print cost (per copy)	0.07	Assumption
	Staff cost	0.033, 0.050, 0.100	Assumption
	Staff payment per person month	443.16	
	Staff working months	6	
	Staff working time (%)	25, 50, 50	
	Number of hospital expanded		
	If vaccination rate increases 10%	In general hospitals (mean = 745, range 500–892)	Assumption
	If vaccination rate increases 15%	In general hospitals and half of the other hospitals (mean = 922, range 627–1102)	
	If vaccination rate increases 25%	All hospitals (mean = 1117, range 768–1319)	

Footnotes: OPTION One includes measures of a single telephone reminder. OPTION Two includes a comprehensive measure of sending a free vaccination voucher and expanding the vaccination sites in hospitals, with one nurse working in each site.

OPTION Two is a comprehensive package consisting of providing vouchers for free vaccination, and expanding vaccination sites to include hospitals. We assumed that this comprehensive measure could increase the vaccination rate by 10%, 15%, and 25%, and the programmatic cost with this measure was assumed to be the same as prior, 1.5 times, and 2.5 times prior cost, respectively.

To meet the increasing demand of vaccination clinics caused by the increase in vaccination rate, we assumed that in OPTION Two, vaccination sites will be expanded in hospitals in the following order: (1) general hospitals for a 10% increase in vaccination coverage rate, (2) further expanded to half of traditional and specialized hospitals for a 15% increase, and (3) to all hospitals for a 25% increase (Table 3) [12]. We assumed there will be one nurse working in each vaccination site.

**Incremental cost effectiveness ratio:** We then used the spreadsheet-based model to calculate the incremental cost effectiveness ratio (ICER) for each option, compared with the current situation of the self-payment policy using the formula below:

ICER (for different options, age groups, and provinces)  =  $ incremental net cost/# incremental cases averted  =  ($ incremental cost of influenza vaccination program – $ incremental cost of health care saved)/(# cases averted by the option – # cases averted in current situation)

### Sensitivity analyses

Although we examined the impact of a wide range of values for a number of critical inputs (Tables 1 and 2), the spreadsheet model allows a user to further explore the impact of changes in input values, either as assumptions or as additional data from the field becomes available.

### Ethics statement

The study was reviewed and approved by the Chinese Center for Disease Control and Prevention Institute Review Board which is registered with the Office for Human Research Protections (active no. in 2012 was IRB 00005183) and has a US Federal Wide Assurance (FWA 00002896). As a study on in-hand surveillance and survey data with no personal contact and no collection of personal data, the Chinese Center for Disease Control and Prevention Institute Review Board waived the need for written informed consent from the individuals.

## Results

### Current situation

The mean (per season) MAC averted for the two age groups (6 to 59 months and 60 months to 14 years) was 1,465 (range: 23∼11,132) and 792 (36∼4,247), respectively, during the studied five influenza seasons (Table 4).

**Table 4:** Cost effectiveness of current influenza vaccination intervention by province and age group in four provinces without subsidy policy in China, comparing with no vaccination.

Age group	Mean # MAC averted (range)	Mean $ of program (range)	Mean $ Net cost(range)	Mean CER for one MAC averted (range)
6–59 ms	1,465(23∼11,132)	6,983(837∼36,209)	−11,430(−111,829∼2,411)	0(−11∼51)
60ms-14yrs	792(36∼4,247)	17,918(2,677∼92,195)	11,965(1,851∼59,412)	37(6∼125)

ms: months; yrs: years

Footnotes:

MAC: medically attended cases including both outpatient and inpatient.

CER: cost-effectiveness ratio. Population number of 6–59 ms and 60 ms-14yrs was 14,301,091 and 45,737,342, respectively.

The mean (range) of influenza vaccination coverage rate of 6–59 ms and 60 ms-14yrs was 11.85% (1.18%–37.69%) and 10.81% (1.18%–39.09%).

Please see Table S4 and S5 in Appendix S1 for data of season and province specific.

The cost of the influenza vaccination program to government for the two age groups was $ 6,983 and $ 17,918, and the CER was $ 0 (−11∼51) and $ 37 (6∼125) per case adverted, respectively (Table 4). For age group 6–59 months, the vaccination program was cost-saving in Henan and Hunan in all studied seasons, in Shandong in season 05/06 and 10/11, and in Sichuan in season 10/11. For age group 60 months to 14 years, it was not cost saving in any season.

### Alternative OPTIONs

**Cost of OPTIONs:** Results for the two alternative strategies are shown in Table 5 and specific seasonal and provincial data are in Table S6 in Appendix S1.

**Table 5:** Example illustration of options to increase influenza vaccination coverage: effectiveness and cost of options comparing with the current situation in four provinces currently without subsidy policy.

Item		OPTION One: telephone reminder			OPTION Two: comprehensive package (free vaccination voucher, and expanding vaccination sites in hospitals)		
Increasing vaccination rate		5%	10%	15%	10%	15%	25%
Additional $ of program per child per dose	Programmatic cost	0.17	0.17	1.015	0.73	0.84	1.18
	Vaccine cost to government	-	-	-	3.95	3.95	3.95
6–59 ms	Mean # MAC averted (range)	6,518(2,355∼11,689)	6,829(2,467∼12,245)	7,139 (2,579∼12,802)	6,829(2,467∼12,245)	7,139(2,579∼12,802)	7,760(2,803∼13,915)
	Mean $ additional Net cost (range)	2,524,821(1,942,053∼3,325,135)	2,522,563(1,936,598∼3,322,974)	1,148,715(888,580∼1,725,849)	8,908,801(7,336,618∼12,935,033)	10,636,205(8,762,536∼15,413,691)	12,045,936(9,287,728∼18,047,836)
60 ms-14 yrs	Mean # MAC averted (range)	3,263(1,921∼4,459)	3,418(2,012∼4,672)	3,574(2,104∼4,884)	3,418(2,012∼4,672)	3,574(2,104∼4,884)	3,884(2,287∼5,309)
	Mean $ additional Net cost (range)	2,568,540(1,980,268∼3,382,730)	2,572,432(1,983,057∼3,388,644)	3,906,746(3,047,917∼5,791,743)	26,113,358(20,810,630-38,346,291)	31,328,683(24,966,635-45,977,245)	40,419,128(31,503,999-59,957,413)

ms: months; yrs: years Footnotes: OPTION One denotes measure of a single recall/reminder to parents by phone. OPTION Two denotes comprehensive measures including providing free influenza vaccination voucher to parents during last visit to EPI clinics and expanding influenza vaccination sites in hospitals. MAC denotes medically attended cases including both outpatient and inpatient. Data of season and province specific please see Table S6 in Appendix S1.

OPTION One would cost an additional $ 0.17 per child per dose for 5% and 10% increased vaccination rate, and $ 1.02 per child per dose for 15% increased vaccination rate. While OPTION Two would require an additional programmatic cost of $ 0.73, $ 0.84, and $ 1.18 for 10%, 15%, and 25% increased vaccination rate, respectively, including the cost of printing vouchers and staff payment for nurses working in the expanded vaccination sites in hospitals (Table 5). OPTION Two also assumes that the government is paying for the vaccine, which means an additional $3.95 per child per dose.

The additional net cost of OPTION One for 5% and 10% influenza vaccination rate increase was more than $ 2.5 million for both age groups. The additional net cost of OPTION One for a 15% increase in the 60 months to 14 years age group was around $ 3.9 million, more than triple that for the 6–59 months age group (approximately $ 1.1 million) (Table 5).

The additional net cost of OPTION Two was much higher than that of OPTION One. The mean additional net cost of OPTION Two was around $ 9–12 million and $ 26–40 million for the 6–59 months and 60 months to 14 years age group, respectively (Table 5).

**Health Outcomes:** The additional numbers of MAC averted by OPTION One for the 6–59 months age group were 6,518 for a 5% increase in influenza vaccination rate, 6,829 for 10% increase, and 7,139 for a 15% increase, around double that of the 60 months to 14 years age group. Similar results were found for numbers of MAC averted by OPTION Two for both age groups (Table 5).

**Figure 2:**
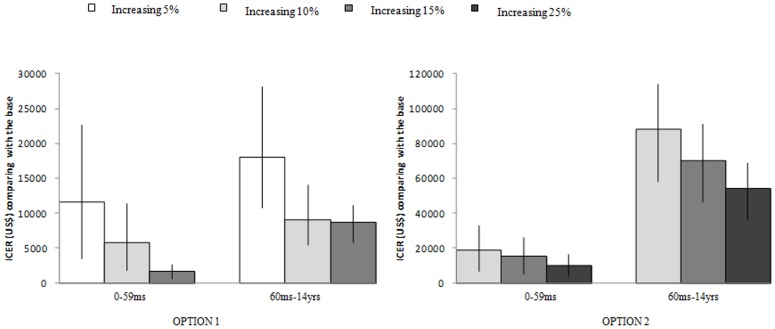
Incremental cost effectiveness ratio (ICER) of two potential strategies to increase influenza vaccination coverage rates among children by age groups in China comparing with the base situation of current policy. OPTION One includes a single telephone vaccination reminder. OPTION Two includes comprehensive measures of free vaccination voucher, and expanding vaccination sites in hospital clinics. The bars in each column shows the ranges due to differences in input values such as rates of influenza like illness (ILI), vaccine effectiveness, and program costs (c.f., Tables 1 and 2).

**Cost Effectiveness:** In comparison with no vaccination, the mean (range) of MAC averted by the current self-payment policy for the two age groups (6 to 59 months and 60 months to 14 years) was 1,465 (23∼11,132) and 792 (36∼4,247), respectively, during the studied five influenza seasons. The total influenza vaccination program cost to government for the two age groups was $ 6,983 and $ 17,918, and the CER was $ 0 (−11∼51) and $ 37 (6∼125) per case adverted, respectively. In comparison with the current policy, the ICER of alternative strategies, OPTION One-telephone reminder and OPTION Two-comprehensive package, decreased as influenza vaccination rate increased. For instance, the highest ICER for OPTION One was $28,118 at a vaccination rate of 5% for children aged 60 months-14 years, but as the vaccination rate increased to 15% the ICER was reduced to $5,838. In OPTION Two, the highest ICER was found to be $113,757 for children 60 months-14 years at a vaccination rate of 10%, but when the vaccination rate increased to 25% the ICER decreased to $36,279. In both OPTIONs, it is concluded that vaccinating children aged 6–59 months is more cost-effective than vaccinating older children (Figure 2). The province specific cost effectiveness of the two OPTIONs is in Table S6 in Appendix S1.

## Discussion

Under the current situation, the influenza vaccination program is more cost-effective among children 6 to 59 months than children 60 months to 14 years old. It costs $0 ($-11∼$51) per case averted among children aged 6–59 months, while it costs $37 ($6∼$125) among children aged 60 months to 14 years old per case averted. Cost-savings was observed among children 6–59 months when the influenza virus was more active and the vaccination rate was relatively high.

The price and demand curve (Figure 1) illustrated that reducing the price of influenza vaccination to parents has a limited effect on increasing the vaccination rate. Alternative strategies are needed in order to increase coverage and thus prevent more influenza cases and related economic loss. Our model was built to demonstrate the potential impact of various policy options.

Both alternative OPTIONs demonstrated in our model had a broad range of ICERs, OPTION One-sending reminder ranged from $590 to $28,118, while OPTION Two-comprehensive measures ranged from $3,753 to $ 113,757.

Switching from the current policy to the alternative OPTION would dramatically increase the vaccination program cost, from $0.03 to more than $5.64 per person per dose at the highest. It requires a significant expenditure. Meanwhile, health care cost saved from additional case averting was relatively low. The estimated cost of influenza outpatient and inpatient was $11.28 and $284.44, which was much lower than that in other countries. Furthermore, the current reimbursement rate of health insurance for children in the four provinces was estimated 19.25% based on a survey [13]. So we anticipate that in places with higher medical cost or better health insurance coverage, an influenza vaccination program will save more health care costs.

The health care utilization pattern revealed in this study may also be the reason for the relatively small number of MAC cases averted. There appeared to be a higher rate of visits to the hospital among patients with severe disease than mild. The rate of outpatients per hospitalization is notably low (i.e. one hospitalized SARI case per 6–9 outpatients) compared to the rate of other countries. For example, in the U.S. the rates of outpatient visits and hospitalizations vary greatly by age and season. The risk of hospitalization for 6–23 month old children, given an outpatient visit, can vary, from one hospitalization for every 18 to 42 outpatient visits. Among older children, where the risk of hospitalization drops, the rate can increase to 52 [26]–[27].

The wide range of ICERs of the two alternative OPTIONs(mean range of two OPTIONs: $1,600∼$87,908) were associated with the large variability in the four provinces, in terms of population size, social economic level, health service utilization, influenza prevalence, influenza vaccination coverage rate, etc. It indicated that in a country with huge disparities like China, decision-making may require more specific data to account for different situations. Our model allows for more precise estimates of influenza prevalence, vaccine effectiveness, disease associated medical costs, and increase in vaccination coverage rate by options and other inputs. It is not limited to the illustrated OPTION One and OPTION Two. It can be a useful tool for evaluation of other alternative strategies, as well as among other populations.

There are a number of limitations to this study, mostly due to lack of available data. We again stress that the objective of this study is to help public health officials assess the value of both investing in such potential programs, as well as collecting additional data to replace the assumptions used here. Although there is vast literature on the epidemiology of influenza, specific data were not always available, thus we had to make some assumptions. For example, to estimate the number of cases, we assumed that outpatient visit (p1) was constant throughout the year. It is possible that there is a seasonal pattern to this value, with an increase during influenza circulation months, which would increase our estimate of influenza cases. On the other hand, we used 24 weeks for the influenza season, which may overestimate the rate of cases seeking medical care for influenza.

Also, the cost of illness from the survey may be biased since study participants came from SARI sentinel surveillance hospitals and most were severe cases. However, it was much lower than the $624 per hospitalized case reported in the Suzhou study [28]. The difference may be influenced by local economics as our study sites are less developed than Suzhou. If hospitalization cost in our model increased to $624 (the governmental cost would increase from $54.67 to $120.12), we would have lower ICERs. It implied that in places with higher economic burden of influenza, the vaccination strategies could be more cost-effective.

Our model is a static model, which does not take into account an intervention's impact upon onwards transmission. This model only incorporates the direct effect of a vaccine, that of protecting an individual from getting sick, whereas a dynamic model (e.g. an ordinary differential equation model) will take into account onwards transmission. Hence, this model is used as a tool to communicate to policy-makers the various options available and their relative impact. Its strengths include the fact that it is relatively simple and easy-to-understand, despite its many limitations.

Furthermore, we used the perspective of the health care system, which only captured the cost and benefit to the health care system. Many studies have shown that lost productivity due to influenza infection among children is substantial [29]; therefore, a more comprehensive societal viewpoint may lead to different cost-effectiveness ratios.

## Conclusion

The spreadsheet-based model we developed is a useful tool in identifying the elements for the cost-effectiveness analysis of using alternative strategies to increase influenza vaccination coverage rate in children. However, to produce more accurate estimates of cost-effectiveness among vaccination policies, more data are needed.

## Supporting Information

Appendix S1 **File includes Tables S1-S4.** Table S1. General information of the five provinces/municipalities. This table provides general information, including areas, overall population, GDP per capita and population aged 6 months to 14 years, of the five provinces (the four studied province Shandong, Henan, Hunan, Sichuan and the compared municipality Beijing). Table S2. Epidemiologic inputs by province, season, and age group. This table showed the epidemiologic inputs that were used to calculate case numbers by province, season and age group from season 05/06 to 10/11, excluding 09/10 the pandemic season. Table S3. Current situation: influenza vaccination coverage rates among target populations by province, season and age group, influenza vaccine effectiveness by season for all ages. This table used data of vaccination coverage rate and effectiveness of influenza vaccine by province from season 05/06 to 11/12, excluding 09/10 the pandemic season. Table S4. Number of influenza cases and cases averted by vaccination program. It showed the calculation results of number of cases and cases averted by the vaccination program, comparing with no vaccination, from season 05/06 to 10/11, excluding 09/10 the pandemic season. Table S5. Current situation: Cost effectiveness of influenza vaccination program in season 05/06-10/11, 09/10 not included; by province, season and age group. Demonstration of results on cost- effectiveness of comparing the current pay-out-of-pocket policy with no vaccination. Table S6. A: cost-effectiveness of OPTION 1-reminder, by province and age group. Demonstration of results on cost-effectiveness of comparing the current situation with two OPTIONS: OPTION 1 reminder and OPTION 2- sending free influenza vaccination voucher and expanding vaccination sites.
(DOCX)

## References

[1] World Health Organization. Vaccine use: WHO recommends annual vaccination for (in order of priority). Available: http://www.who.int/influenza/vaccines/use/en/. Accessed 14 June 2013.

[2] Centers for Disease Control and Prevention. Seasonal Influenza (Flu): Recommendations of the Advisory Committee on Immunization Practices (ACIP). Available: http://www.cdc.gov/flu/professionals/acip/index.htm. Accessed 14 June 2013.

[3] van Essen GA, Palache AM, Forleo E, Fedson DS (2003) Influenza vaccination in 2000: recommendations and vaccine use in 50 developed and rapidly developing countries. Vaccine 21: 1780–1785. 12686094 10.1016/s0264-410x(03)00072-0

[4] Ministry of Health of People's Republic of China. Guideline on seasonal influenza vaccination. Available: http://www.moh.gov.cn/publicfiles/business/htmlfiles/mohbgt/pw10303/200804/18615.htm. Accessed 14 June 2013.

[5] Chinese Center for Disease Control and Prevention (2007) Guideline on seasonal influenza vaccination during the 2007–2008 season in China; 2007. Zhong Hua Liu Xing Bing Xue Za Zhi 28: 1144–1145.

[6] Chinese Center for Disease Control and Prevention(2008). Guideline on seasonal influenza vaccination during the 2008–2009 season in China; Available: http://www.hbzjcdc.com/main/guifanfangan/200810/641.htm.Accessed 14 June 2013.

[7] Chinese Center for Disease Control and Prevention(2009) Guideline on seasonal influenza vaccination during the 2009–2010 season in China; Available: http://www.gszhuanglang.gov.cn/jkzx/wzgg/content_4567_13689.Accessed 14 June 2013.

[8] Chinese Center for Disease Control and Prevention(2010)Guideline on seasonal influenza vaccination during the 2010–2011 season in China. Available: http://www.chinacdc.cn/tzgg/201011/t20101124_40513.htm. Accessed 14 June 2013.

[9] Feng L, Mounts AW, Feng Y, Luo Y, Yang P, et al (2010) Seasonal influenza vaccine supply and target vaccinated population in China, 2004-2009. Vaccine 28: 6778–6782 10.1016/j.vaccine.2010.07.064. Epub 2010 Aug 3 20688038

[10] National Bureau of Statistics of China (2010) China statistical yearbook 2010. Beijing: China Statistics Press.

[11] Liang W, Feng L, Xu C, Xiang N, Zhang Y, et al (2012) Response to the first wave of pandemic (H1N1) 2009: Experiences and lessons learnt from China. Public Health 126: 427–436 10.1016/j.puhe.2012.02.008. Epub 2012 Apr 18 22516790 PMC7111655

[12] Ministry of Health of People's Republic of China (2008) The 4^th^ National Health Service Survey.

[13] Zhou L, Situ S, Huang T, Hu S, Wang X, et al (2013) Direct Medical Cost of Influenza-Related Hospitalizations among Severe Acute Respiratory Infections Cases in Three Provinces in China. PLoS ONE 8(5): e63788 10.1371/journal.pone.0063788 23717485 PMC3661662

[14] Chinese Center for Disease Control and Prevention (2011). SARI surveillance protocol [in Chinese]. Available: http://www.chinacdc.cn/jkzt/crb/lxxgm/jszl_2251/201111/t20111117_54776.htm. Accessed 31 January 2013.

[15] Zhou L, Su Q, Xu Z, Feng A, Jin H, et al (2013) Seasonal Influenza Vaccination Coverage Rate of Target Groups in Selected Cities and Provinces in China by Season (2009/10 to 2011/12). PLoS ONE 8(9): e73724 10.1371/journal.pone.0073724 24040041 PMC3767785

[16] Monto AS, Ohmit SE, Petrie JG, Johnson E, Truscon R, et al (2009) Comparative Efficacy of Inactivated and Live Attenuated Influenza Vaccines. N Engl J Med 361: 1260–1267 10.1056/NEJMoa0808652 19776407

[17] Skowronski DM, De Serres G, Dickinson J, Petric M, Mak A, et al (2009) Component-specific effectiveness of trivalent influenza vaccine as monitored through a sentinel surveillance network in Canada, 2006–2007. J Infect Dis 199: 168–179 10.1086/595862 19086914

[18] Skowronski DM, Masaro C, Kwindt TL, Mak A, Petric M, et al. (2007) Estimating vaccine effectiveness against laboratory-confirmed influenza using a sentinel physician network: Results from the 2005–2006 season of dual A and B vaccine mismatch in Canada. Vaccine 25: :2842–2851. Epub 2006 Oct 16. 10.1016/j.vaccine.2006.10.002 17081662

[19] Hak E, Hermens RP, Hoes AW, Verheij TJ, Kuyvenhoven MM, et al (2000) Effectiveness of a co-ordinated nation-wide programme to improve influenza immunisation rates in the Netherlands. Scand J Prim Health Care 18: 237–241. 11205093 10.1080/028134300448814

[20] Steens A, van der Hoek W, Dijkstra F, van der Sande M. (2011) Influenza vaccine effectiveness, 2010/11. Euro Surveill16(15): :pii = 19843. Available online: http://www.eurosurveillance.org/ViewArticle.aspx?ArticleId=19843. 21507318

[21] Yu W, Yu J, Cui G, Jin S, Wang J, et al (2006) Study on the reasonable cost of national immunization program in some regions of China. Chinese J of Vaccines and Immunization 4: 280–284.

[22] Beijing Center for Disease Control and Prevention. News: Elder people and school children can receive free influenza vaccination in Beijing. Available: http://www.bjcdc.org/news.php?id=36641. Accessed 14 June 2013.

[23] Rancé F, Chave C, de Blic J, Deschildre A, Donato L, et al (2008) Influenza vaccination coverage in asthmatic children in Fance in 2006–2007 (article in French). Arch Pediatr 11: 1724–1728. 10.1016/j.arcped.2008.09.004 19090033

[24] Daley MF, Barrow J, Pearson K, Crane LA, Gao D, et al (2004) Identification and Recall of Children With Chronic Medical Conditions for Influenza Vaccination. Pediatrics 113: e26–e33. 14702491 10.1542/peds.113.1.e26

[25] Britto MT, Schoettker PJ, Pandzik GM, Weiland J, Mandel KE (2007) Improving influenza immunisation for high-risk children and adolescents. Qual Saf Health Care 16: 363–368 10.1136/qshc.2006.019380 17913778 PMC2464966

[26] Meltzer MI, Neuzil KM, Griffin MR, Fukuda K (2005) An economic analysis of annual influenza vaccination of children. Vaccine 23: 1004–1014. 15620473 10.1016/j.vaccine.2004.07.040

[27] Prosser LA, Bridges CB, Uyeki TM, Hinrichsen VL, Meltzer MI, et al (2006) Health benefits, risks, and cost-effectiveness of influenza vaccination of children. Emerg Infect Dis 12: 1548–1558 10.3201/eid1210.051015. 17176570 PMC3290928

[28] Zhang T, Zhu Q, Zhang X, Ding Y, Steinhoff M, et al (2012) The Clinical Characteristics and Direct Medical Cost of Influenza in Hospitalized Children: A Five-Year Retrospective Study in Suzhou, China. PLoS ONE 7(9): e44391 10.1371/journal.pone.0044391 22957069 PMC3434134

[29] Szucs T (1999) The socio-economic burden of influenza J Antimicrob Chemother 44 Suppl B:11–15. 10.1093/jac/44.suppl_2.11 10877457

