# Supplementary material for: Cost-Effectiveness of Alternative Strategies for Annual Influenza Vaccination among Children Aged 6 Months to 14 Years in Four Provinces in China
Source: PLoS One. 2014 Jan 31;9(1):e87590. doi: 10.1371/journal.pone.0087590 (PMC3909220; doi:10.1371/journal.pone.0087590)
Supplement: Appendix S1 — File includes Tables S1-S4. Table S1. General information of the five provinces/municipalities. This table provides general information, including areas, overall population, GDP per capita and population aged 6 months to 14 years, of the five provinces (the four studied province Shandong, Henan, Hunan, Sichuan and the compared municipality Beijing). Table S2. Epidemiologic inputs by province, season, and age group. This table showed the epidemiologic inputs that were used to calculate case numbers by province, season and age group from season 05/06 to 10/11, excluding 09/10 the pandemic season. Table S3. Current situation: influenza vaccination coverage rates among target populations by province, season and age group, influenza vaccine effectiveness by season for all ages. This table used data of vaccination coverage rate and effectiveness of influenza vaccine by province from season 05/06 to 11/12, excluding 09/10 the pandemic season. Table S4. Number of influenza cases and cases averted by vaccination program. It showed the calculation results of number of cases and cases averted by the vaccination program, comparing with no vaccination, from season 05/06 to 10/11, excluding 09/10 the pandemic season. Table S5. Current situation: Cost effectiveness of influenza vaccination program in season 05/06-10/11, 09/10 not included; by province, season and age group. Demonstration of results on cost- effectiveness of comparing the current pay-out-of-pocket policy with no vaccination. Table S6. A: cost-effectiveness of OPTION 1-reminder, by province and age group. Demonstration of results on cost-effectiveness of comparing the current situation with two OPTIONS: OPTION 1 reminder and OPTION 2- sending free influenza vaccination voucher and expanding vaccination sites. (DOCX) [file pone.0087590.s001.docx]

**Supplementary Appendices**

**Cost-effectiveness of alternative strategies for annual influenza vaccination among children aged 6 months to 14 years in four provinces in China**

Lei Zhou^1^, Sujian Situ^2^, Zijian Feng^1*^, Charisma Y. Atkins^3^, Isaac Chun-Hai Fung^3,4^, Zhen Xu^5^, Ting Huang^6^, Shixiong Hu^7^, Xianjun Wang^8^, Martin I. Meltzer^3^

^1^ Public Health Emergency Center, Chinese Center for Disease Control and Prevention, Beijing, China

^2^ U.S. Centers for Disease Control and Prevention, Beijing, China

^3^ U.S. Centers for Disease Control and Prevention, National Center for Emerging and Zoonotic Infectious Diseases, Division of Preparedness and Emerging Infections, Atlanta, GA, United States of America

^4^ Department of Epidemiology, Jiann-Ping Hsu College of Public Health, Georgia Southern University, Statesboro, GA, United States of America

^5^ Key Laboratory of Surveillance and Early-warning on Infectious Disease, Division of Infectious Disease, Chinese Center for Disease Control and Prevention, Beijing, China

^6^ Sichuan Center for Disease Control and Prevention, Chengdu, Sichuan, China

^7^ Hunan Center for Disease Control and Prevention, Changsha, Hunan, China

^8^ Shandong Center for Disease Control and Prevention, Jinan, Shandong, China

^*^ Corresponding author

Supplementary Table S1: General information of the five provinces/municipalities

| Province/Municipality | area, square km | population | GDP per capital (USD) | Population (6-14 years) | |
| --- | --- | --- | --- | --- | --- |
|  |  |  |  | 6-59 months | 5-14 years |
| Beijing | 16,410.54 | 20,186,000 | 10,328 | 401,842 | 1,285,158 |
| Shandong | 157,100 | 95,793,065 | 7006 | 3,590,682 | 11,483,618 |
| Henan | 167,000 | 94,020,000 | 4279 | 4,703,459 | 15,042,467 |
| Hunan | 211,800 | 71,193,400 | 4074 | 2,756,857 | 8,816,900 |
| Sichuan | 485,000 | 89,981,705 | 3195 | 3,250,094 | 10,394,356 |
| Total | 1,037,310.54 | 371,174,170 | - | 14,702,934 | 47,022,499 |

Source:

Population: China Statistic yearbook 2010 and estimation based on the ratio from WHO/Immunization country profile

Area and GDP: The Central People’s Government of The People’s Republic of China, http://www.gov.cn

Note: Exchange rate=6.77

Supplementary Table S2: Epidemiologic inputs by province, season, and age group Data Source

| Input | Definition of inputs | Province | age group |  | Influenza seasons | | | | Source |
| --- | --- | --- | --- | --- | --- | --- | --- | --- | --- |
|  |  |  |  | 05/06 | 06/07 | 07/08 | 08/09 | 10/11 |  |
| p1 | No. of outpatient consultation persons per 100 persons with illness onset within 2 weeks (including all cause illness) | National | 6-59ms | 297.60 | 297.60 | 297.60 | 297.60 | 297.60 | ref # 12 |
|  |  |  | 60 ms-14 yrs | 109.20 | 109.20 | 109.20 | 109.20 | 109.20 |  |
| p2 | No. of ILI per 100 outpatient of age specific | Shandong | 6-59 ms | 1.27 | 0.82 | 0.64 | 0.81 | 0.82 | China CDC ILI sentinel surveillance data |
|  |  |  | 60 ms-14 yrs | 0.76 | 0.39 | 0.29 | 0.41 | 0.45 |  |
|  |  | Henan | 6-59 ms | 1.72 | 2.62 | 2.95 | 2.24 | 1.25 |  |
|  |  |  | 60 ms-14 yrs | 0.66 | 0.68 | 0.99 | 0.75 | 0.50 |  |
|  |  | Hunan | 6-59 ms | 2.40 | 2.59 | 2.93 | 2.49 | 3.30 |  |
|  |  |  | 60 ms-14 yrs | 0.79 | 0.46 | 0.72 | 0.85 | 0.79 |  |
|  |  | Sichuan | 6-59 ms | 0.62 | 0.22 | 0.27 | 0.38 | 1.30 |  |
|  |  |  | 60 ms-14 yrs | 0.42 | 0.19 | 0.16 | 0.22 | 0.53 |  |
| p3 | No. of influenza positive per 100 ILI with sample collection and lab test of age group specific | | 6-59 ms | 9.26 | 8.88 | 9.03 | 6.95 | 11.30 | China CDC ILI sentinel surveillance data |
|  |  |  | 60 ms-14 yrs | 22.72 | 16.15 | 17.18 | 16.52 | 14.77 |  |
| p4 | Median No. of ILI among outpatients in SARI sentinel hospital per 100 SARI hospitalization in same SARI sentinel hospital of age specific | | 6-59 ms | 563.00 | 563.00 | 563.00 | 563.00 | 563.00 | China CDC SARI sentinel surveillance data |
|  |  |  | 60 ms-14 yrs | 858.00 | 858.00 | 858.00 | 858.00 | 858.00 |  |
| p5 | No. of influenza positive per 100 SARI case of age specific | | 6ms-14 yrs | 14.97 | 14.97 | 14.97 | 14.97 | 14.97 | China CDC SARI sentinel surveillance data |

ms: months; yrs: yearsSupplementary Table S3: Current situation: influenza vaccination coverage rates among target populations by province, season and age group, influenza vaccine effectiveness by season for all ages

| Name | Province | Age Group | Seasons | | | | | | Data Source |
| --- | --- | --- | --- | --- | --- | --- | --- | --- | --- |
|  |  |  | 05/06 | 06/07 | 07/08 | 08/09 | 10/11 | 11/12 |  |
| The influenza vaccination coverage rate (VCR) among target population (%) | Beijing | 6-59 ms | 5.06 | 3.97 | 6.92 | 17.90 | 26.10 | 15.81 | VCR of season 05-09 are assumed as ratio of sales vs population, and VCR of two age groups are assumed the same; VCR of 10/11 and 11/12 were from a survey by China CDC in 2011 |
|  |  | 60 ms-14yrs | 5.06 | 3.97 | 6.92 | 17.90 | 47.70 | 26.32 |  |
|  | Shandong | 6-59 ms | 2.24 | 2.35 | 3.84 | 3.87 | 25.40 | 22.46 |  |
|  |  | 60 ms-14yrs | 2.24 | 2.35 | 3.84 | 3.87 | 26.90 | 14.80 |  |
|  | Henan | 6-59 ms | 2.45 | 2.32 | 1.90 | 2.41 | 37.69 | 29.97 |  |
|  |  | 60 ms-14yrs | 2.45 | 2.32 | 1.90 | 2.41 | 39.09 | 23.86 |  |
|  | Hunan | 6-59 ms | 1.18 | 1.22 | 3.37 | 3.32 | 37.30 | 33.44 |  |
|  |  | 60 ms-14yrs | 1.18 | 1.22 | 3.37 | 3.32 | 37.20 | 22.76 |  |
|  | Sichuan | 6-59 ms | 1.65 | 1.77 | 4.53 | 3.03 | 31.44 | 25.33 |  |
|  |  | 60 ms-14yrs | 1.65 | 1.77 | 4.53 | 3.03 | 34.69 | 18.62 |  |
| Effectiveness of influenza vaccine | national | all age | 63.00% | 52.00% | 68.00% | 61.00% | 61.00% | 61.00% | ref # 13 |
|  |  | 95% CI | 15-84% | 23-70% | 46-81% | NA | NA | NA |  |

ms: months; yrs: years

Supplementary Table S4: Number of influenza cases and cases averted by vaccination program

S4-a: Number of outpatient cases and cases averted by vaccination program

| Age group | Province | 05/06 | | 06/07 | | 07/08 | | 08/09 | | 10/11 | |
| --- | --- | --- | --- | --- | --- | --- | --- | --- | --- | --- | --- |
|  |  | #case | #averted | #case | #averted | #case | #averted | #case | #averted | #case | #averted |
| 6-59ms | Shandong | 12567 | 180 | 7781 | 96 | 6176 | 166 | 6016 | 146 | 9902 | 1815 |
|  | Henan | 22294 | 349 | 32566 | 398 | 37287 | 487 | 21791 | 325 | 19771 | 5903 |
|  | Hunan | 18233 | 137 | 18869 | 120 | 21707 | 509 | 14198 | 293 | 30594 | 9011 |
|  | Sichuan | 5553 | 58 | 1890 | 18 | 2358 | 75 | 2554 | 48 | 14209 | 3372 |
| 60ms-14yrs | Shandong | 21653 | 310 | 7898 | 98 | 6248 | 168 | 8494 | 205 | 8335 | 1636 |
|  | Henan | 24632 | 386 | 18039 | 221 | 27938 | 365 | 20352 | 303 | 12131 | 3798 |
|  | Hunan | 17281 | 130 | 7153 | 46 | 11910 | 279 | 13520 | 279 | 11234 | 3298 |
|  | Sichuan | 10831 | 114 | 3483 | 32 | 3120 | 99 | 4125 | 78 | 8885 | 2385 |

S4-b: Number of inpatient cases and cases averted by vaccination program

| Age group | Province | 05/06 | | 06/07 | | 07/08 | | 08/09 | | 10/11 | |
| --- | --- | --- | --- | --- | --- | --- | --- | --- | --- | --- | --- |
|  |  | #case | #averted | #case | #averted | #case | #averted | #case | #averted | #case | #averted |
| 6-59ms | Shandong | 3609 | 52 | 2330 | 29 | 1818 | 49 | 2301 | 56 | 2330 | 427 |
|  | Henan | 6402 | 100 | 9751 | 119 | 10980 | 143 | 8337 | 124 | 4652 | 1389 |
|  | Hunan | 5236 | 39 | 5650 | 36 | 6392 | 150 | 5432 | 112 | 7199 | 2120 |
|  | Sichuan | 1595 | 17 | 566 | 5 | 694 | 22 | 977 | 18 | 3343 | 793 |
| 60ms-14yrs | Shandong | 1663 | 24 | 853 | 11 | 635 | 17 | 897 | 22 | 985 | 193 |
|  | Henan | 1892 | 30 | 1949 | 24 | 2837 | 37 | 2150 | 32 | 1433 | 449 |
|  | Hunan | 1327 | 10 | 773 | 5 | 1209 | 28 | 1428 | 30 | 1327 | 390 |
|  | Sichuan | 832 | 9 | 376 | 3 | 317 | 10 | 436 | 8 | 1050 | 282 |

ms: months; yrs: years

Supplementary Table S5: Current situation: Cost effectiveness of influenza vaccination program in season 05/06-10/11, 09/10 not included; by province, season and age group

| Age group | Province | 05/06 | | | | 06/07 | | | | 07/08 | | | | 08/09 | | | | 10/11 | | | |
| --- | --- | --- | --- | --- | --- | --- | --- | --- | --- | --- | --- | --- | --- | --- | --- | --- | --- | --- | --- | --- | --- |
|  |  | $  health care saved | $ pro-gram | net cost | $/case adver-ted | $  health care saved | $ pro-gram | net cost | $/case adver-ted | $  health care saved | $ pro-gram | net cost | $/case adver-ted | $  health care saved | $ pro-gram | net cost | $/case adver-ted | $  health care saved | $ pro-gram | net cost | $/case adver-ted |
| 6-59 ms | Shandong | 3219 | 2069 | -1150 | -5 | 1785 | 2168 | 383 | 3 | 3029 | 3546 | 517 | 2 | 3360 | 3571 | 211 | 1 | 27299 | 20716 | -6583 | -3 |
|  | Henan | 6236 | 2955 | -3281 | -7 | 7384 | 2807 | -4577 | -9 | 8897 | 2290 | -6606 | -10 | 7502 | 2910 | -4592 | -10 | 88762 | 36209 | -52553 | -7 |
|  | Hunan | 2445 | 837 | -1608 | -9 | 2234 | 864 | -1370 | -9 | 9292 | 2384 | -6908 | -10 | 6774 | 2350 | -4424 | -11 | 135510 | 23681 | -111829 | -10 |
|  | Sichuan | 1042 | 1377 | 335 | 4 | 325 | 1475 | 1150 | 51 | 1368 | 3779 | 2411 | 25 | 1110 | 2528 | 1418 | 21 | 50700 | 21147 | -29553 | -7 |
| 60 ms-14 yrs | Shandong | 1977 | 6616 | 4639 | 14 | 790 | 6933 | 6144 | 57 | 1296 | 11341 | 10045 | 54 | 1633 | 11422 | 9789 | 43 | 14121 | 43657 | 29536 | 16 |
|  | Henan | 2456 | 9452 | 6995 | 17 | 1782 | 8976 | 7194 | 29 | 2818 | 7324 | 4506 | 11 | 2411 | 9305 | 6894 | 21 | 32782 | 92195 | 59412 | 14 |
|  | Hunan | 826 | 2677 | 1851 | 13 | 369 | 2763 | 2394 | 47 | 2155 | 7624 | 5469 | 18 | 2220 | 7516 | 5296 | 17 | 28461 | 51547 | 23086 | 6 |
|  | Sichuan | 724 | 4403 | 3679 | 30 | 261 | 4718 | 4457 | 125 | 765 | 12086 | 11321 | 104 | 617 | 8086 | 7469 | 87 | 20584 | 49716 | 29132 | 11 |

Supplementary Table S6: A: cost-effectiveness of the twoOPTIONS

6-A: Cost-effectiveness of OPTION 1-reminder; by province and age group

| Age group | Province | 5% | | | | | 10% | | | | | 15% | | | | |
| --- | --- | --- | --- | --- | --- | --- | --- | --- | --- | --- | --- | --- | --- | --- | --- | --- |
|  |  | △case averted | △health care $ saved | △cost of program | △Net Cost | ICER | △case averted | △health care $ saved | △cost of program | △Net Cost | ICER | △case averted | △health care $ saved | △cost of program | △Net Cost | ICER |
| 6-59 ms | Shandong | 112 | 1365 | 2536864 | 2535499 | 22612 | 224 | 2730 | 2538036 | 2535306 | 11305 | 336 | 4095 | 892675 | 888580 | 2642 |
|  | Henan | 365 | 4438 | 3329573 | 3325135 | 9120 | 729 | 8876 | 3331850 | 3322974 | 4557 | 1094 | 13314 | 1739164 | 1725849 | 1578 |
|  | Hunan | 557 | 6775 | 1948828 | 1942053 | 3489 | 1113 | 13551 | 1950149 | 1936598 | 1740 | 1670 | 20326 | 1006158 | 985831 | 590 |
|  | Sichuan | 208 | 2535 | 2299132 | 2296597 | 11028 | 416 | 5070 | 2300445 | 2295375 | 5511 | 625 | 7605 | 1002204 | 994599 | 1592 |
| 60 ms-14 yrs | Shandong | 91 | 706 | 2572641 | 2571935 | 28118 | 183 | 1412 | 2576609 | 2575197 | 14077 | 274 | 2118 | 3050035 | 3047917 | 11107 |
|  | Henan | 212 | 1639 | 3384370 | 3382730 | 15930 | 425 | 3278 | 3391922 | 3388644 | 7979 | 637 | 4917 | 5796661 | 5791743 | 9092 |
|  | Hunan | 184 | 1423 | 1981691 | 1980268 | 10742 | 369 | 2846 | 1985903 | 1983057 | 5378 | 553 | 4269 | 3233223 | 3228954 | 5838 |
|  | Sichuan | 133 | 1029 | 2340257 | 2339228 | 17545 | 267 | 2058 | 2344889 | 2342830 | 8786 | 400 | 3088 | 3561455 | 3558368 | 8896 |

S6-B: cost-effectiveness of OPTION 2- sending free influenza vaccination voucher and expanding vaccination sites

| Age group | Province | 10% | | | | | 15% | | | | | 25% | | | | |
| --- | --- | --- | --- | --- | --- | --- | --- | --- | --- | --- | --- | --- | --- | --- | --- | --- |
|  |  | △case averted | △health care $ saved | △cost of program | △Net Cost | ICER | △case averted | △health care $ saved | △cost of program | △Net Cost | ICER | △case averted | △health care $ saved | △cost of program | △Net Cost | ICER |
| 6-59 ms | Shandong | 224 | 2730 | 7339348 | 7336618 | 32715 | 336 | 4095 | 8766631 | 8762536 | 26049 | 561 | 6825 | 9294553 | 9287728 | 16566 |
|  | Henan | 729 | 8876 | 12943909 | 12935033 | 17739 | 1094 | 13314 | 15427005 | 15413691 | 14092 | 1823 | 22190 | 18070026 | 18047836 | 9901 |
|  | Hunan | 1113 | 13551 | 7538036 | 7524485 | 6759 | 1670 | 20326 | 9005400 | 8985074 | 5381 | 2783 | 33877 | 10479182 | 10445305 | 3753 |
|  | Sichuan | 416 | 5070 | 7844137 | 7839067 | 18821 | 625 | 7605 | 9391126 | 9383521 | 15020 | 1041 | 12675 | 10415550 | 10402875 | 9991 |
| 60 ms-14 yrs | Shandong | 183 | 1412 | 20812042 | 20810630 | 113757 | 274 | 2118 | 24968753 | 24966635 | 90983 | 457 | 3530 | 31507530 | 31503999 | 68884 |
|  | Henan | 425 | 3278 | 38349569 | 38346291 | 90291 | 637 | 4917 | 45982163 | 45977245 | 72172 | 1062 | 8196 | 59965608 | 59957413 | 56471 |
|  | Hunan | 369 | 2846 | 21467753 | 21464907 | 58216 | 553 | 4269 | 25757972 | 25753703 | 46565 | 922 | 7115 | 33448357 | 33441242 | 36279 |
|  | Sichuan | 267 | 2058 | 23833665 | 23831606 | 89370 | 400 | 3088 | 28620238 | 28617150 | 71544 | 667 | 5146 | 36779002 | 36773856 | 55162 |
